# Combined Radiation and Endocrine Therapies Elicit Benefit in ER+ Breast Cancer

**DOI:** 10.3390/cancers17121921

**Published:** 2025-06-09

**Authors:** Anneka L. Johnson, Steven Tau, Austin M. Sloop, Tianyuan Dai, Alyssa M. Roberts, Patricia Muskus, Alexa Warren, Sierra A. Kleist, Riley A. Hampsch, Julie M. Jorns, Rongxiao Zhang, Lesley A. Jarvis, Todd W. Miller

**Affiliations:** 1 Department of Molecular and Systems Biology, Dartmouth Geisel School of Medicine, Lebanon, NH 03766, USA; 2 Thayer School of Engineering, Dartmouth College, Hanover, NH 03755, USA; 3 Department of Radiation Oncology, Dartmouth Geisel School of Medicine, Lebanon, NH 03766, USA; 4 Department of Pharmacology and Toxicology, Medical College of Wisconsin, Milwaukee, WI 53226, USA; 5 Department of Pathology, Medical College of Wisconsin, Milwaukee, WI 53226, USA; 6 Department of Medical Physics, University of Missouri School of Medicine, Columbia, MO 65201, USA

**Keywords:** breast cancer, endocrine therapy, antiestrogen, radiation, oxidative stress, drug-tolerant persisters

## Abstract

Background: Standard treatment for patients with early-stage estrogen receptor-positive (ER+) breast cancer often includes sequential adjuvant radiation and endocrine therapies. Unfortunately, ~1/3 of patients eventually experience disease recurrence, partly due to residual disease in the form of drug-tolerant persister cancer cells. The anti-cancer efficacy of radiation therapy is partly attributable to the production of oxyradicals that damage biomolecules. We previously showed that endocrine therapy increases mitochondrial content in ER+ breast cancer cells; we postulated that this may also increase oxidative stress. Methods: Herein, we tested the efficacy of concurrent endocrine and radiation therapies, including both conventional (CDR) and ultra-high dose rate (UHDR) radiation. Results: We found that estrogen deprivation and radiation inhibit cell growth, induce apoptosis, and force cells into an oxidatively stressed state. DNA damage was almost exclusive to cells treated with the combination of endocrine and radiation therapy. Radiation slowed tumor growth in two xenograft models, and combination with estrogen deprivation prolonged the time to regrowth in ZR75-1 tumors. Conclusions: These findings indicate that simultaneous treatment with endocrine and radiation therapies can be advantageous, warranting further evaluation to identify tumor features predictive of response to individual and combination treatments.

## Simple Summary

Recurrence remains a major challenge for a significant proportion of estrogen receptor-positive breast cancer patients. The purpose of this study is to exploit the oxidative stress phenotype induced by radiation and endocrine therapies through concurrent use. We established that oxidative stress is induced by both therapies and can be advantageous in combination at inducing apoptosis and slowing tumor growth. These results provide rationale for further investigation into leveraging oxidative stress and discovering markers predictive of efficacy.

## 1. Introduction

Breast cancer (BC) is the most frequently diagnosed cancer in women, with over 300,000 new cases annually in the United States. This cancer type is also the leading cause of non-smoking-related cancer mortality [1]. Approximately 2/3 of BC cases express estrogen receptor α (ER) [2]. The standard of care for patients with Stage I-III ER+ BC is surgical removal of the primary tumor(s) and adjacent lymph node(s), frequently followed by adjuvant radiation therapy (RT) to the breast and surrounding tumor-involved tissue [3]. After completion of a course of RT, patients are treated with systemic endocrine therapy for ≥5 years to reduce the transcriptional activity of ER, a major cancer driver in this disease [4,5]. (Neo)adjuvant chemotherapy can also be used as a treatment for patients with ER+ BC considered at high risk of recurrence [6]. Neoadjuvant endocrine therapy is also used as a bridge to surgery in patients experiencing a delay in surgery.

Despite the aforementioned treatment modalities, not all cancer cells in the body are consistently eradicated, leaving behind “drug-tolerant persister” (DTP) cancer cells that survive treatment and may ultimately cause recurrent disease. Approximately 1/3 of patients (~400,000 women annually worldwide) with early-stage disease eventually experience cancer recurrence, which is often more difficult to treat, frequently metastatic, and usually ultimately lethal [7,8,9]. The latency period between initial BC diagnosis and disease recurrence can be long (>20 years) [10], suggesting that DTPs can undergo long periods of survival without detectable outgrowth. This latency provides a window of opportunity to treat DTPs and prevent their outgrowth.

Herein, we report that ER+ BC DTPs surviving endocrine therapy exhibit an increased oxidative state, which we posited could act as a radiosensitizer. Conventional RT delivered at a dose rate of 0.03 to 0.1 Gy/s results in toxicity to both cancer and non-cancer cells, eliciting adverse effects such as hair loss, skin damage, and fibrosis. The anti-cancer efficacy of conventional dose rate (CDR) RT is thought to be at least partially due to the induction of oxyradicals that damage DNA, resulting in cell death by cell cycle arrest and eventual apoptosis or mitotic catastrophe [11]. Recently developed ultra-high dose rate RT (≥40 Gy/s) has been shown to elicit similar anti-cancer efficacy as CDR RT with reduced toxicity to normal tissues; this is known as the “FLASH effect” [12,13]. We tested the effects of combined endocrine therapy and RT on ER+ BC cells and tumors to determine the translational potential of such combined therapy in the (neo)adjuvant setting.

## 2. Materials and Methods

### 2.1. Cell Culture and Irradiation

MCF-7, T47D, and ZR75-1 cells were obtained from the American Type Culture Collection (ATCC). Cells were maintained in DMEM supplemented with 10% FBS (R&D Systems, Minneapolis, MN, USA). Cells were stably transfected with lentivirus encoding luciferase-GFP, followed by puromycin (1 µg/mL) selection as in ref. [14]. Hormone deprivation (HD) involved culture in phenol red-free DMEM containing 10% dextran/charcoal-stripped FBS (DCC-FBS; R&D Systems) and 2 mM Glutamax (ThermoFisher Scientific, Waltham, MA, USA). Cells pre-treated with HD were treated with 6 Gy conventional (CDR) or ultra-high dose rate (UHDR) radiation using a Varian Linac-based/modified beam; radiochromic film (EBT-XD) was used to match UHDR and conventional doses. Cell lines were confirmed to be mycoplasma-free (Universal Mycoplasma Detection Kit; ATCC) and authenticated by STR genotyping (University of Vermont Cancer Center DNA Analysis Facility).

### 2.2. Growth Assays

Cells were seeded in triplicate at 2000 cells/well for MCF-7 and T47D cells, and 5000 cells/well for ZR75-1 cells in 12-well plates. In growth assays with HD, cells were seeded and then washed and treated with HD medium for the indicated time in a staggered fashion so wells could all be endpointed simultaneously after 21 d in culture. Irradiated cells were treated with 6 Gy at conventional or ultra-high dose rates and then allowed to grow for 21 d prior to the endpoint. At the endpoint, cells were fixed and stained with 0.5% crystal violet in 20% methanol for 10 min. Cells were rinsed with water, and plates were allowed to dry. Plates were scanned using a LiCor Odyssey M, and the area of staining was measured using ImageJ (version v1.54p) (RRID:SCR_003070).

### 2.3. Western Blot

Cells were lysed in RIPA buffer (20 mmol/L Tris, pH 7.4, 1 mmol/L EDTA, 1 mmol/L EGTA, 150 mmol/L NaCl, 50 mmol/L NaF, 5 mmol/L NAPPi, 10 mmol/L Na b-glycerophosphate, 10% glycerol, 1% NP-40) with a Protease Inhibitor Cocktail (Millipore-Sigma, Burlington, MA, USA) and 1 nmol/L Na_3_VO_4_ (New England Biolabs, Ipswich, MA, USA) on ice for 10 min. Cell lysates were then scraped, sonicated with a Qsonica Q125 sonicator at 30% power for 15 s, and centrifuged at 21,000× *g* for 10 min at 4 °C. Protein concentration was measured with a BCA Assay (Pierce, Rockford, IL, USA), and the results were utilized to standardize protein concentration across samples. Lysates were denatured and reduced using 4× LDS sample buffer (GenScript, Piscataway, NJ, USA) with 1.25% b-mercaptoethanol. Proteins were separated by SDS-PAGE and transferred to a nitrocellulose membrane before Ponceau S staining for visual confirmation of protein transfer. Membranes were probed with primary antibodies for NRF2 (Cell Signaling Technology, Danvers, MA, USA #12721 or GeneTex, Irvine, CA, USA, #GTX103322), vinculin (Cell Signaling Technology #13901), or b-actin (Cell Signaling Technology #3700) overnight at 4 °C. Secondary antibodies conjugated to horseradish peroxidase (GE Healthcare, Marlborough, MA, USA) were utilized to detect signal, and blots were developed using ECL substrate (Pierce #32106). Signal was visualized using ChemiDoc MP (Bio-Rad, Des Plaines, IL, USA) or ImageQuant 800 (Cytiva Amersham, Little Chalfont, Amersham HP7 9NA, UK).

### 2.4. Antioxidant Intracellular State Measurement In Vitro

T47D and MCF-7 cells underwent HD as indicated (0–30 d), re-seeded in 96-well plates (5000 cells/well), and irradiated ± 6 Gy using a Varian Linac-based/modified beam. Following radiation, the medium was changed, and cells were assayed 3 d later according to the manufacturer’s instructions for the GSH/GSSG-Glo Assay (Promega, Madison, WI, USA, #V6611) or NADP/NADPH-Glo Assay (Promega #G9081). Luminescent signals were measured using a plate reader (BioRad, iMark Microplate Reader).

### 2.5. In Vitro Apoptosis Assay

Cells underwent HD as indicated and then were re-seeded (~150,000 cells/well) in 12-well plates. Cells were then treated with ± 6 Gy radiation the following day. The medium was immediately changed, and 3 d later, cells were assayed for apoptosis using a TACS Annexin V-FITC Apoptosis Detection Kit (R&D Systems #4830-250-K) per the manufacturer’s instructions. Stained cells were analyzed by flow cytometry on a MACSQuant (Miltenyi, Auburn, CA, USA), and data were analyzed with FlowJo software (version 10.10.0) (BD Biosciences, Franklin Lakes, NJ, USA).

### 2.6. Mouse Studies

Animal studies were approved by the Institutional Animal Care and Use Committee review board at Dartmouth College (protocol 2144). Female NOD-scid/IL2Rγ^−/−^ (NSG) mice (from Dartmouth Mouse Modeling Shared Resource) were injected bilaterally with 5–10 million MCF-7/Luc or ZR75-1/Luc BC cells into mammary fat pads and s.c. implanted with a beeswax pellet containing 1 mg 17β-estradiol (E2) [15]. Tumor dimensions were measured twice weekly with calipers, and volume was calculated as (length × width^2^)/2. When the volume of a tumor reached 200 mm^3^, mice were randomized to treatment arms. Prior to treatment, a CT scan was taken of a representative mouse for each tumor model to help inform radiation beam delivery to the tumor and avoid vital organs. A subset of mice was used for weekly bioluminescence imaging: mice were injected i.p. with 100 µL of in vivo-grade D-Luciferin (3 mg/mL; Promega) in PBS and placed under isoflurane anesthesia; after a 10 min uptake period, mice were imaged for bioluminescence using a Xenogen IVIS 200 System; bioluminescent signal values were analyzed using Living Image software (version 4.8.2) (Perkin Elmer, Springfield, IL, USA). Estrogen deprivation (ED) was achieved by removing the E2 pellet. CDR RT and UHDR RT were administered to isoflurane-anesthetized mice as single sessions of 16 Gy to each tumor using a Varian Trilogy Linear Accelerator or Mobetron Intraoperative Linear Election Accelerator (IntraOP). The Varian Trilogy Linear Accelerator was modified to allow the irradiator to deliver 10 MeV ultra-high dose rate radiation (UHDR RT) while maintaining conventional capabilities. Modifications made to the Varian allow for the use of the higher electron gun currents and higher pulse repetition rates offered by photon energy modes, but with the X-ray target removed from the beamline to grant access to a high-fluence 10 MeV electron beam. Mechanical and pneumatic adjustments also allow for rapid switching between CDR and UHDR modes [16]. UHDR treatments under the Trilogy consisted of 14 × 4 µs pulses delivered at 360 Hz (443 Gy/s MDR) to the machine isocenter with the electron applicator accessory and a 1 cm brass final aperture. CDR RT was delivered at the same geometry using 10 Gy/minute electron dose rates. UHDR treatments under the Mobetron were set up with a 1.6 cm Cerrobend aperture installed in the faceplate position, with 4 × 3.13 µs pulsed delivered at 90 Hz (704 Gy/s MDR). CDR RT groups were treated at the conventional geometry with a 1.6cm diameter circular aperture. Five mm of bolus was used for all treatments. Surgical tape was used to stretch the hind limb and hold it away from the abdomen to minimize radiation dose to vital organs. To help restrict the radiation field to a tumor area, a 1.6 cm^2^ circular shielded collimator was used. Dose delivery was calibrated by measuring the UHDR radiation dose with radiochromic film (EBT-XD) and a thermoluminescent dosimeter. The CDR radiation dose was then matched to the UHDR dose as measured with radiochromic film. Film was also used between animal treatments with CDR and UHDR RT to confirm that the beam output was within 5% of the target value. For molecular analysis, tumors were harvested 24 h after irradiation, and tumor fragments were formalin-fixed and paraffin-embedded (FFPE) or snap-frozen in liquid nitrogen.

### 2.7. Immunohistochemistry

FFPE tissue was cut into 5 µm sections and mounted on slides. Slides were deparaffinized in xylene and rehydrated with a graded ethanol series. Antigen retrieval was performed in citrate buffer, pH 6 (Sigma-Aldrich, St. Louis, MO, USA), in a pressure cooker at high pressure for 20 min. Sections were then permeabilized with 0.2% Triton X-100 in PBS. Sections were blocked by incubating in 5% goat serum (in Vectastain ABC-HRP Kit from Vector Labs, Newark, CA, USA, #PK-4002; RRID: AB_2336811) in TBS with 0.1% Tween-20 (TBS-T) for 1 h followed by overnight incubation with primary antibody in blocking solution at 4 °C. Primary antibodies included NRF2 (GeneTex #GTX103322; RRID:AB_1950993), Ki67 (Leica Biosystems, Nusslock, Germany, clone MM1 #PA0410-U; RRID:AB_563842), and ER (Ventana Medical Systems/Roche Tissue Diagnostics, Tucson, AZ, USA, clone SP1 #790-4325; RRID. AB_10981779). Slides were washed with TBS-T, treated with 0.3% hydrogen peroxide, and the signal was developed using VectaStain ABC-HRP Kit. Sections were counterstained with hematoxylin and Scott’s solution. Sections were dehydrated with an ethanol series, washed with xylene, and sealed with coverslips. NRF2 staining intensity was quantified from 5 representative microscopic fields of view using CellProfiler software (version 4.2.6) (RRID:SCR_007358). Ki67 and ER staining were visually quantified and reviewed by a fellowship-trained breast pathologist by conventional clinical methods [17,18].

### 2.8. Immunofluorescence

Cell lines: Cells were pre-treated with HD as indicated, re-seeded onto glass coverslips, and then irradiated with 6 Gy CDR RT or UHDR RT. At 16 h after radiation, cells were fixed with 1.85% formaldehyde for 10 min and permeabilized with 0.1% Triton X-100 in PBS for 10 min. After washing with TBS-T, cells were blocked with 1% BSA in TBST for 1 h at room temp. Cells were probed with primary antibody (phospho-histone H2AX_S139_, Cell Signaling Technology #9718; RRID: AB_2118009) in blocking buffer overnight at 4 °C on a rocker. Cells were washed with TBS-T, and the signal was amplified with fluorescent secondary antibodies (goat-anti-rabbit Alexa Fluor 488, ThermoFisher Scientific, catalog # A11029, RRID: AB_2534088) for 1 h in the dark at room temp. Cells were washed in TBS-T and PBS, and coverslips were mounted on slides with ProLong Gold Antifade Mountant with DNA Stain DAPI (ThermoFisher Scientific #P36935). Cells were imaged at 400× magnification using a Zeiss LSM 800 confocal microscope. Foci per nucleus in ≥100 nuclei per condition were quantified using CellProfiler software (RRID: SCR_007358).

Tumors: Five-micron sections of FFPE tissue were mounted on slides, deparaffinized in xylene, and rehydrated as for the immunohistochemistry methods. Tissues were blocked in 5% BSA with 0.3% Triton X-100 in PBS for 1 h, washed in 3% BSA in PBS, and probed with primary antibody (phospho-histone H2AX_S139_, Cell Signaling Technologies, Danvers, MA, USA #80312, RRID:AB_2799949) in 1% BSA with 0.3% Triton X-100 in PBS at 4 °C overnight. Tissues were then washed with PBS and incubated with secondary antibodies (goat-anti-mouse Alexa Fluor 647, ThermoFisher Scientific #A21235, RRID: AB_2535804) in 1% BSA with 0.3% Triton X-100 in PBS for 1.5 h at room temperature. Slides were washed with PBS, treated with TrueVIEW Autofluorescence Quenching Kit (Vector Labs #SP-8400-15), and mounted in ProLong Glass Antifade Mountant with NucBlue Stain (ThermoFisher Scientific #P36985). Slides were imaged on a Leica SP8 upright confocal microscope. Staining was quantified using CellProfiler software.

### 2.9. Oxidative Stress Response Score

We generated an oxidative stress response (OSR) R-score using 39 oxidative phosphorylation (OXPHOS) signature genes derived from MCF-7 cells treated with hydrogen peroxide, menadione, and tert-butyl hydroperoxide [19]. The signatures CHUANG_OXIDATIVE_STRESS_RESPONSE_DN and CHUANG_OXIDATIVE_STRESS_RESPONSE_UP were obtained from the Molecular Signatures Database [20] and contained 11 and 28 genes, respectively. Gene expression values for the 39 signature genes were extracted from published RNA sequencing datasets in the NCBI Gene Expression Omnibus (GSE20181, GSE71791, GSE111563) that include transcriptomic profiles from primary ER+ breast tumor specimens acquired before and after presurgical endocrine therapy. Spearman correlation analysis was used to calculate the correlation (R-score) between tumor gene expression profiles and the OXPHOS signature as in ref [21].

### 2.10. Statistics

Cell growth data, OSR R-scores, and IHC scores were analyzed by *t*-test (for 2-group experiments), or ANOVA followed by Bonferroni multiple comparison-adjusted post hoc testing between groups (for experiments with ≥3 groups). Tumor volumes were analyzed by the mixed-effects model with Bonferroni adjustment for multiple comparisons using Prism software (GraphPad, version 10.5.0.774).

## 3. Results

### 3.1. Estrogen Deprivation Slows Growth and Increases Oxidative Stress Response

**Figure 1:**
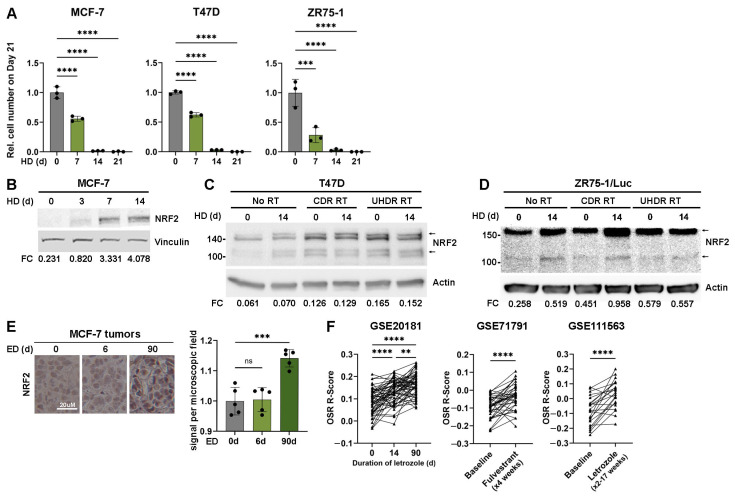
Endocrine therapy slows growth and induces oxidative stress in ER+ BC cells. (**A**) Cells were seeded in triplicate and allowed to grow for 21 d. Cells underwent HD for the indicated time frame immediately prior to analysis. (**B**) Cells underwent HD as indicated, and lysates were analyzed by immunoblot. (**C**,**D**) Cells were maintained in growth medium or HD for 14 d prior to being treated with 6 Gy CDR or UHDR radiation. Lysates were harvested 1 h after radiation and analyzed by immunoblot. (**E**) Orthotopic xenografts were established in ovariectomized NSG mice supplemented with E2 by s.c. pellet. E2 pellets were removed from tumor-bearing mice for 0, 6, or 90 d to induce ED. Tumors and residual tumor beds were harvested and analyzed by IHC. Signal intensity in five microscopic fields was measured per tumor. (**F**) A gene expression signature of oxidative stress response (OSR) was compared with transcriptional profiles of human tumors sampled before and after presurgical endocrine therapy in 3 patient cohorts. Spearman correlation R values were calculated as correlation coefficients between the OSR signature and tumor gene expression values. In (**A**,**E**), data are presented as mean ± SD. ** *p* ≤ 0.01, *** *p* ≤ 0.001, **** *p* ≤ 0.0001 by Bonferroni-adjusted post hoc test ((**A**,**E**), and cohort GSE20181 in (**F**)) or paired *t*-test (cohorts GSE71791 and GSE111563 in (**F**)). Original blot images can be found in Supplementary File S1.

The estrogen deprivation (ED) induced by aromatase inhibitors in patients can be mimicked in vitro with hormone deprivation (HD) of cultured cells. Increasing length of HD progressively inhibited the growth of MCF-7, T47D, and ZR75-1 ER+ BC cells (Figure 1A). Oxidative stress disrupts kelch-like ECH-associated protein 1 (KEAP1)-mediated degradation of nuclear factor erythroid 2-related factor 2 (NRF2), which allows NRF2 accumulation. Therefore, an increase in NRF2 expression indicates that cells are oxidatively stressed [22,23]. In MCF-7 cells, HD for ≥3 d induced NRF2 upregulation (Figure 1B). This phenomenon remained consistent in T47D and ZR75-1/Luc cells evaluated after 14 d of HD (Figure 1C,D).

To evaluate whether estrogen deprivation induces oxidative stress in vivo, ovariectomized NSG mice were injected with MCF-7 cells and implanted with a slow-release E2 pellet to induce tumor formation. We previously reported that these estrogen-driven tumors regress to become non-palpable after several weeks of ED [14]. When a tumor reached 200 mm^3^, the E2 pellet was removed, and tumors were harvested after 0, 6, or 90 days after ED. Immunohistochemical analysis showed that NRF2 expression was significantly increased after 90 d of ED in residual tumor cells (Figure 1E). To measure how ED affects oxidative state in human tumors, we analyzed transcriptional profiles from early-stage ER+ BC specimens treated with presurgical endocrine therapy (letrozole or fulvestrant) in three clinical studies [24,25,26]. Using a 39-gene oxidative stress response (OSR) signature generated from MCF-7 cells [19], we calculated an OSR R-score for each tumor specimen that reflects correlation with the OSR signature. Tumors in all three clinical studies showed increased OSR R-scores upon treatment with endocrine therapy (Figure 1F). Therefore, endocrine therapy increases signs of oxidative stress in cell lines, xenografts in mice, and patient tumors.

### 3.2. Metabolic Analysis Indicates an Increased Oxidative State Due to Hormone Deprivation and Radiation

**Figure 2:**
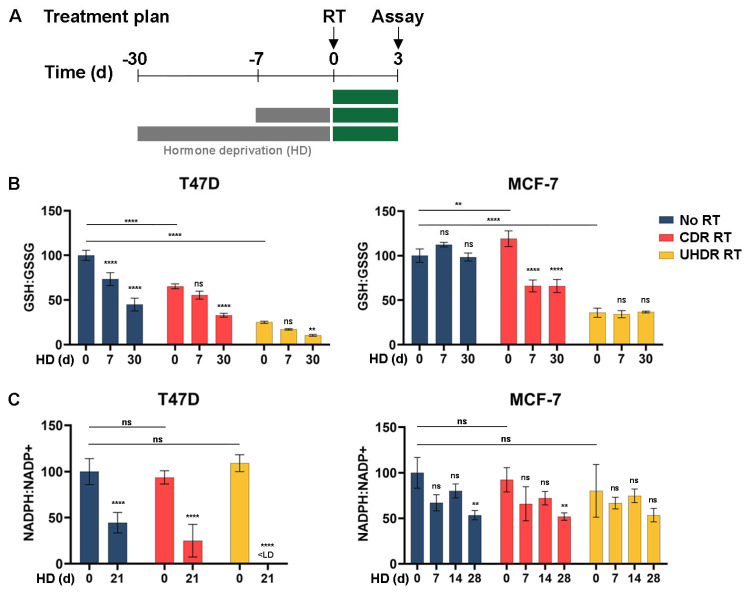
Oxidative state increases upon hormone deprivation and irradiation in ER+ BC cells. (**A**) Treatment plan overview. (**B**) Cells underwent HD as indicated and then were treated with ±6 Gy CDR RT or UHDR RT. At 72 h post-RT, relative levels of total and reduced glutathione were measured, which are shown as the ratio of reduced glutathione (GSH) to oxidized glutathione (GSSG). (**C**) Cells were treated as in (**B**). Relative levels of total and reduced NADP were measured, which are shown as the ratio of reduced NADP (NADPH) to oxidized NADP (NADP+). < LD: below limit of detection. Data are presented as mean of triplicate ± SD. ** *p* ≤ 0.01, **** *p* ≤ 0.0001 by Bonferroni-adjusted post hoc test compared to respective Day 0 group unless otherwise indicated with brackets. ns: not significant; FC: fold change represented by the ratio for NRF2 to Actin signal measured via ImageJ.

Reactive oxygen species (ROS) can damage biomolecules and disrupt cellular functions. Cells use metabolic buffering mechanisms to neutralize ROS, including glutathione and NADPH [27,28,29,30]. Given the increase in oxidative stress markers in response to endocrine therapy (Figure 1), we analyzed the levels of oxidized and reduced glutathione and nicotinamide adenine dinucleotide phosphates (NADP) in vitro. HD decreased the ratios of reduced/oxidized glutathione and NADP in T47D cells, and decreased the reduced/oxidized NADP ratio in MCF-7 cells (Figure 2), both of which indicate an increased oxidative state caused by HD.

RT is postulated to elicit anti-cancer effects in part through the generation of ROS [31]. Indeed, elevated NRF2, a marker of oxidative stress, was observed at 1 h after irradiation of cultured cells with 6 Gy using CDR RT or UHDR dose rates. This effect potentially saturated NRF2 levels in T47D cells, where the addition of HD did not elicit further effects (Figure 1C,D). As measured at 72 h post-RT, CDR RT and UHDR RT decreased the ratio of reduced/oxidized glutathione but not NADP in T47D cells, and UHDR RT decreased the reduced/oxidized glutathione ratio in MCF-7 cells (Figure 2). Differences between these metabolic markers may be partly attributable to differences in metabolic buffering during the post-radiation period. The addition of HD exacerbated the oxidative state induced by RT in T47D cells. In MCF-7 cells, the combination of HD and CDR RT increased glutathione oxidation, but UHDR RT may have maximally induced glutathione oxidation that was not further enhanced by HD (Figure 2B,C).

### 3.3. Radiation Inhibits Cell Growth and Hormone Deprivation Induces Apoptosis

**Figure 3:**
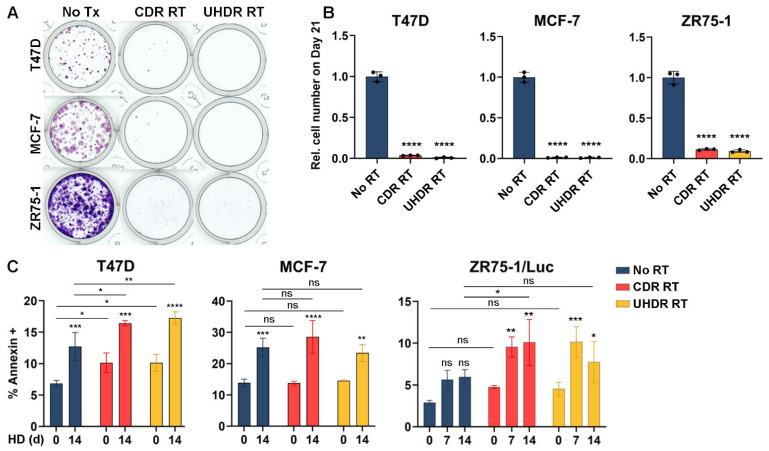
Radiation inhibits growth and HD apoptosis in ER+ BC cells. (**A**,**B**) Cells were treated ±6 Gy CDR RT or UHDR RT and then maintained for 21 d. Representative images are shown in (**A**). Quantification of relative cell numbers is shown in (**B**). (**C**) Cells underwent HD as indicated and then were treated ± RT as above. Medium was changed, and 3 d later, cells were analyzed for apoptosis by annexin V labeling and flow cytometry. Data are presented as mean of triplicate ± SD. * *p* ≤ 0.05, ** *p* ≤ 0.01, *** *p* ≤ 0.001, **** *p* ≤ 0.0001 by Bonferroni-adjusted post hoc test compared to respective “Day 0” group unless otherwise indicated. ns: not significant.

Based on our observations that RT increases the oxidative state (Figure 1 and Figure 2), we tested the effects of RT on cell growth in hormone-replete conditions. Treatment with 6 Gy of RT at CDR or UHDR dose rates robustly suppressed cell growth (Figure 3A,B). To determine whether RT induced apoptosis, we measured the proportion of cells exhibiting surface annexin V positivity in a 3-day window following RT. RT induced apoptosis in T47D cells but not in other cell lines (Figure 3C). HD of cells for 14 d increased apoptosis in T47D and MCF-7 cells, which was slightly increased further by RT in T47D cells. Combination treatment with HD followed by RT induced apoptosis of ZR75-1 cells, while either treatment alone did not (Figure 3C). Propidium iodide uptake was also evaluated, but only a small proportion of cells (<3%) were PI-positive/annexin-negative, and proportions were similar among treatment conditions.

### 3.4. Combined Hormone Deprivation and Irradiation Induces DNA Damage

**Figure 4:**
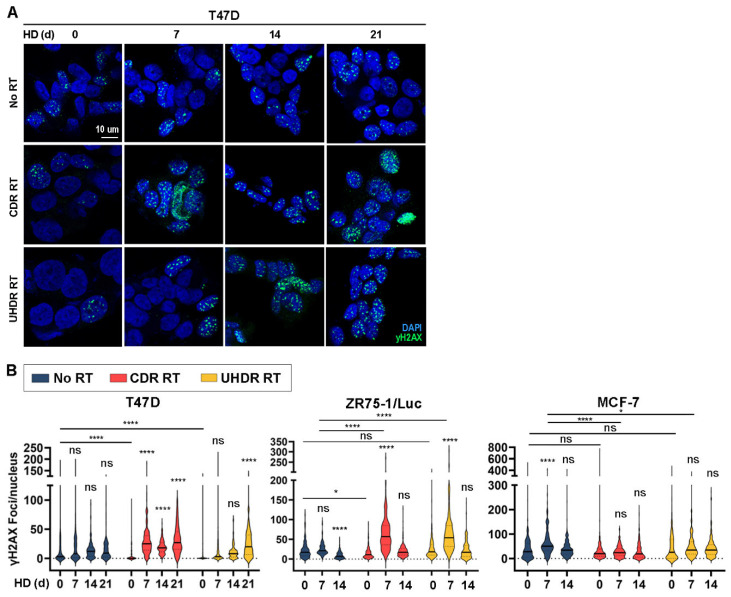
Combination treatment with hormone deprivation and radiation induces DNA damage in ER+ BC cells. Cells underwent HD as indicated and then were treated ±6 Gy CDR RT or UHDR RT. After 16 h, cells were immunostained for γH2AX and counterstained with DAPI. (**A**) Representative images are shown. (**B**) Quantification of γH2AX foci per nucleus in ≥100 cells/group. Data are presented as violin plots. * *p* ≤ 0.05, **** *p* ≤ 0.0001 by Bonferroni-adjusted post hoc test compared to respective Day 0 group unless otherwise indicated with brackets. ns: not significant.

We considered that if HD and RT each increase oxidative state, then the consequent increased ROS may drive DNA damage [32,33]. Immunostaining for phospho-H2AX_S139_ (γH2AX) as a marker of DNA damage [34] showed no major changes in accumulation as foci (reflecting sites of DNA breaks) at 16 h after RT. HD decreased γH2AX focus formation in ZR75-1/Luc cells and transiently increased γH2AX foci in MCF-7 cells (Figure 4). The most drastic increases in γH2AX foci occurred after combined treatment with HD followed by RT in T47D and ZR75-1/Luc cells. However, differences between cell lines in the effects of HD duration and RT on γH2AX focus formation suggest that the DNA-damaging effects of these treatments may not be generalizable across ER+ BC.

### 3.5. Co-Treatment with Hormone Deprivation and Radiation Is Sometimes Advantageous in Tumor Growth Studies Across Multiple Models

**Figure 5:**
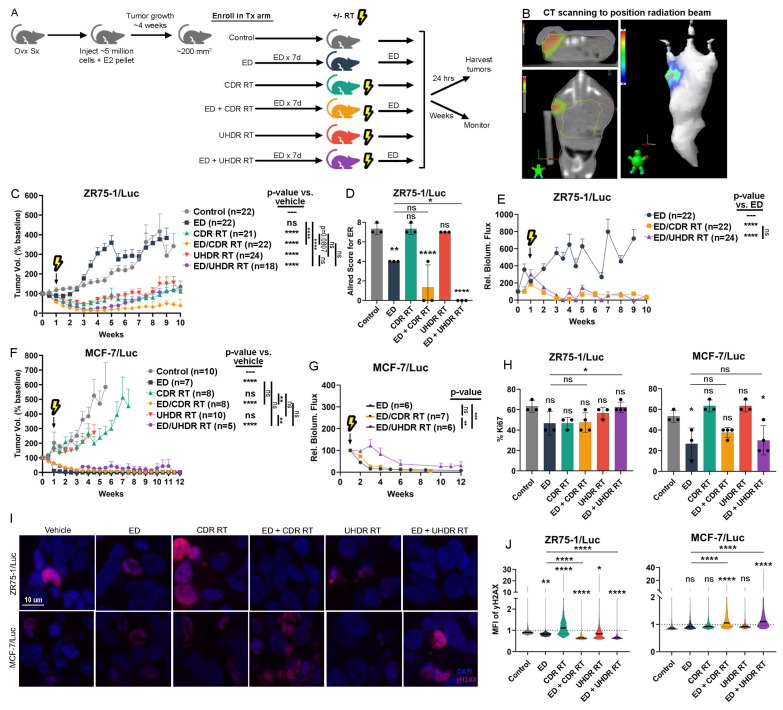
Radiation and endocrine therapies combine to prevent tumor growth. (**A**) Treatment plan overview. Ovariectomized mice with E2 supplementation bearing tumors underwent ± ED for 7 d and then ± CDR RT or UHDR RT. (**B**) Representative images from a CT scan of a mouse bearing an MCF-7/Luc tumor, which was used to help aim the radiation beam. (**C**) ZR75-1/Luc tumor growth curves displayed as percent change from tumor volume at study enrollment. (**D**) Allred score of ER IHC staining from tumors (n = 3/group) harvested at the end of the experiment in (**C**). (**E**) Quantification of relative flux (p/s) of bioluminescence images from tumors in (**C**). (**F**) MCF-7/Luc tumor growth curves displayed as in (**C**). (**G**) Quantification of relative flux (p/s) of bioluminescence images from tumors in (**F**). (**H**) Percent positivity of Ki67 IHC staining of tumors (n ≥ 3/group) harvested 24 h after RT. (**I**) Representative images and (**J**) quantification of ICC staining for γH2AX from tumors (n ≥ 3/group, ≥1200 nuclei/group) harvested 24 h after RT. Data are shown as mean ± SD, except tumor growth curves are shown with SEM (C/F). * *p* ≤ 0.05, ** *p* ≤ 0.01, *** *p* ≤ 0.001, **** *p* ≤ 0.0001 by Bonferroni-adjusted post hoc test compared to vehicle (**D**), and by mixed-effects model with Bonferroni adjustment for multiple comparisons (**C**,**E**–**G**).

We tested the effects of endocrine therapy and RT on tumor xenografts in vivo. Ovariectomized NSG mice were s.c. implanted with an E2 pellet and injected with ZR75-1/Luc cells. E2 deprivation (ED) via the removal of the E2 pellet was used to mimic the effects of aromatase inhibition in humans. Mice bearing tumors that reached 200 mm^3^ were randomized to treatment ± ED for 7 d, followed by treatment ± one fraction of 16 Gy RT at CDR or UHDR dose rates (Figure 5A). ED was initiated 7 d before RT to allow time for ER signaling to subside and oxidative state to increase. CT scanning was performed on a representative animal before each RT session to aid in radiation beam positioning (Figure 5B).

Control-treated mice with continuous E2 supplementation showed continued tumor growth (Figure 5C and Figure S1A). Although ED transiently slowed tumor growth, tumors quickly became E2-independent as reflected by their rapid time to recurrence (defined as regrowth to baseline volume, Figure S1B). Immunohistochemical analysis revealed decreased ER levels of ED-treated tumors (±RT) (Figure 5D), suggesting that ER loss may be associated with endocrine resistance in this model system. CDR and UHDR RT each induced tumor regression followed by regrowth in the presence of continued E2 supplementation (both *p* < 0.0001 compared to control). Combination treatment with ED and RT (at CDR and UHDR dose rates) also induced tumor regression (both *p* < 0.0001 compared to control). The addition of ED to CDR RT showed a trend towards improved efficacy (*p* = 0.0807) compared to CDR RT alone, but such an effect of ED was not observed in the context of UHDR RT (Figure 5C). The combination of ED/CDR RT significantly delayed tumor recurrence compared to ED/UHDR RT (Figure S1B). Bioluminescence imaging of a subset of mice showed similar results: tumors in ED-treated mice exhibited increased bioluminescence over time, indicating tumor growth, and combination treatments decreased bioluminescence with no statistically significant difference between types of RT (Figure 5E).

In the MCF-7/Luc xenograft model shown to be estrogen-dependent [14], ED significantly induced tumor regression (Figure 5F and Figure S1C). In contrast to effects seen in ZR75-1/Luc tumors (Figure 5C), RT at either a CDR or UHDR dose rate did not significantly affect MCF-7/Luc tumor growth compared to control in the context of continued E2 supplementation (Figure 5F). The combination of ED and RT similarly induced MCF-7/Luc tumor regression. We performed bioluminescence imaging to resolve differences in residual tumor burden: the addition of either CDR or UHDR RT to ED more rapidly decreased tumor burden than ED alone (Figure 5G).

Immunohistochemical analysis of ZR75-1/Luc tumors harvested 24 h after RT revealed no significant differences in the proliferation marker Ki67 in each treatment group compared to control (Figure 5H). However, ED/UHDR RT combination-treated tumors showed slightly higher proportions of Ki67+ cells than ED-treated tumors. In contrast, MCF-7/Luc tumors from the three treatment groups that regressed (ED, ED/CDR RT, and ED/UHDR RT) trended towards a reduction in Ki67 positivity, but RT alone had no significant effect (Figure 5H). Immunofluorescence analysis of γH2AX showed that only CDR RT significantly induced DNA damage in ZR75-1/Luc tumors, while other treatments reduced DNA damage (Figure 5I,J). In MCF-7/Luc tumors, only the combination treatments with ED and RT increased γH2AX staining compared to either control or ED.

## 4. Discussion

Metabolic vulnerabilities, including oxidative stress, offer targets for cancer therapy [35,36,37,38]. We report for the first time that endocrine therapy increased oxidative stress in ER+ BC cells, xenografts, and patient tumors (Figure 1 and Figure 2). Since RT also increased oxidative stress (Figure 1C,D and Figure 2), we postulated that combined endocrine and radiation therapies would elicit superior anti-cancer effects. These treatments significantly inhibited growth in vitro (Figure 1A and Figure 3A,B) with variable contributions to DNA damage (Figure 4) and apoptosis (Figure 3C) across cell lines. While the additional anti-tumor effects of RT were obscured by a dramatic response to ED in estrogen-driven MCF-7 tumors, RT induced the regression of ZR75-1 tumors that were proven to be estrogen-independent (Figure 5C,F). These findings collectively suggest that endocrine therapy can elicit radiosensitizing effects in ER+ BC, but such effects vary between biological models.

We found that HD increased oxidative stress in ER + BC models (in vitro and in vivo) and primary human tumors (Figure 1B–F and Figure 2). Hormone signaling has also been shown to modulate oxidative stress in other systems. In the ovary, a delicate balance of ROS is maintained to aid oocyte maturation. As estrogen levels decline with age, ROS levels become less regulated, leading to ROS accumulation and oxidative stress that results in DNA damage and oocyte death [39]. Prostate cancer cell growth is frequently driven by androgen receptor activation. Evidence shows that androgen deprivation via castration or pharmacological inhibition increases ROS in prostate cancer cells. Castration-resistant prostate cancer showed the downregulation of SOD2, which in turn increases ROS; this increased ROS induced androgen receptor activation and promoted resistance to anti-androgen therapy. Confirming the role of oxidative stress in this mechanism, treatment with the antioxidant N-acetylcysteine (NAC) partly prevented those effects [40,41,42]. The induction of oxidative stress has also been observed with other anti-cancer agents targeting the PI3K/AKT/mTOR, MEK/ERK, and STAT3 signaling axes, although effects differ between model systems [43,44]. Therefore, oxidative stress-related pathways may be modulated indirectly using existing anti-cancer agents.

We show that radiation increases apoptosis by approximately 10% in T47D and ZR75-1 cells (Figure 3C), while long-term growth assays showed near-complete growth inhibition due to radiation treatment (Figure 3A,B). In addition to apoptosis, other cell death mechanisms may contribute to growth inhibition. Prior evidence suggests that radiation induces ferroptosis through upregulation of ACSL4, which is responsible for the biosynthesis of polyunsaturated fatty acid-containing phospholipids. The ablation of ACSL4 eliminates radiation-induced ferroptosis [45]. Examining the role of ferroptosis in radiation- and HD-induced cell death is an avenue for further study.

RT is thought to exert anti-cancer effects in part through the induction of oxyradicals that damage biomolecules in cells and eventually cause apoptosis. We found that RT increases oxidative stress in ER+ BC cells (Figure 1C,D and Figure 2). Radiation induces ROS directly and/or indirectly by the radiolysis of water inside cells [46,47]. Increased ROS due to RT can result in DNA damage that is detrimental to cell survival [31,48]. While RT induced modest degrees of apoptosis in T47D cells (Figure 3C), RT alone either did not induce or modestly suppressed DNA damage in our study (Figure 4). The effects of RT on the induction of DNA damage and apoptosis were more pronounced in the context of HD in both T47D and ZR75-1 models, suggesting that the induction of such DNA damage occurs despite decreased cell cycling elicited by HD. HD and RT also decreased glutathione levels (Figure 2B), and there is evidence that suppressing the production of buffering metabolites like glutathione prevents DNA damage repair, contributing to the efficacy of RT [49]. Such reduced buffering of ROS may underlie the increased DNA damage observed in vitro and in vivo (Figure 4A,B and Figure 5I,J). Michmerhuizen et al. also found that the treatment of MCF-7 or T47D cells with the anti-estrogens tamoxifen or fulvestrant enhanced the growth-suppressive effects of RT [50]. Interestingly, these antiestrogens elicited different degrees of enhancement of RT effects based on the timing of drug administration, possibly due in part to the use of the pro-drug tamoxifen rather than its active metabolites 4-hydroxy-tamoxifen or endoxifen. Fulvestrant treatment at 6 h post-RT elicited growth-suppressive effects akin to those of fulvestrant administered at 1–24 h pre-RT, suggesting that ER inhibition within this time window surrounding RT can be effective. Combined endocrine/radiation therapy additively increased DNA breakage and triggered senescence in their study.

In contrast to findings described herein and in ref. [50], other in vitro studies reported that treatment with tamoxifen prior to or concurrent with RT reduces the efficacy of RT [51,52,53]. A proposed rationale is that tamoxifen prevents cell cycling, and the consequent slowing of DNA replication is radioprotective. Another study found that tamoxifen does not change radiosensitization [54]. Two retrospective analyses of clinical studies showed that even though there is a theoretical challenge to concurrent treatment with tamoxifen and RT, theory did not translate into practice: treatment with concurrent tamoxifen and RT did not compromise overall or disease-free survival in patients [55,56].

A limitation of our in vivo study is that mice were ED for only 7 d prior to the irradiation of tumors (Figure 5). Similarly, Michmerhuizen et al. administered tamoxifen to mice bearing MCF-7 xenografts on Days 0–10, and administered RT on either Days 1–6 or 6–10; with both schedules, tamoxifen sensitized tumors to RT (administered at 2 Gy/min once daily for 5 d), providing near-stasis of tumor volumes for >3 months [50]. We showed that robust oxidative stress (as indicated by NRF2 accumulation) is induced after 90 d of ED (Figure 1E). Therefore, a longer period of ED prior to RT could drive tumor cells into a more oxidatively stressed state that could be better exploited by RT; a future study may test the effects of such longer-term ED on radiosensitization.

## 5. Conclusions

In agreement with studies demonstrating that UHDR RT has similar anti-tumor efficacy compared to CDR RT (e.g., ref. [12]), we found that these dose rates elicited similar anti-tumor effects in vivo (Figure 5). However, UHDR RT more effectively induced glutathione oxidation in cultured cells than CDR RT (Figure 2B). Treatment with ED did not significantly affect mouse body weight (Figure S2). Although RT prevented body weight gain, combination treatment with RT and ED did not have such effects. In summary, the results herein show that combined RT and endocrine therapy induce oxidative stress, DNA damage, apoptosis, and growth-suppressive effects on ER+ BC cells and tumors in mice. However, the effects of this combined therapy varied between biological models, suggesting that there are underlying features that dictate sensitivity; further analysis to identify such features may yield biomarkers to predict the benefits. In addition to targeting oncogenic endocrine signaling in steroid hormone receptor-driven cancer types, evidence suggests that targeting alternative oncogenic pathways can elicit radiosensitizing effects. In hepatocellular carcinoma, treatment with the PI3K inhibitor BKM120 improves the efficacy of RT and induces apoptosis [57]. MDM2 regulates the tumor suppressor p53; in an adenoid cystic carcinoma PDX model, MDM2 inhibition with AMG 232 combined with RT enabled complete tumor regression [58]. Therefore, the inhibition of oncogenic signaling, despite consequent slowing of cell cycle progression, enhances response to RT across cancer types and provides impetus to study the broader effects of such agents on radiosensitization to identify a unifying mechanism.

## Data Availability

The data presented in this study are available in this article and Supplementary Materials.

## Supplementary Materials

The following supporting information can be downloaded at: https://www.mdpi.com/article/10.3390/cancers17121921/s1, Figure S1: Individual tumor growth curves for in vivo studies; Figure S2: Body weights of mice bearing ZR75-1/Luc tumors; File S1: Original blot images.
