# Supplementary material for: Combined Radiation and Endocrine Therapies Elicit Benefit in ER+ Breast Cancer
Source: Cancers (Basel). 2025 Jun 9;17(12):1921. doi: 10.3390/cancers17121921 (PMC12190792; doi:10.3390/cancers17121921)
Supplement: Supplementary file 1 [file cancers-17-01921-s001.zip › Figures S1 and S2.pdf]

Supplementary Data

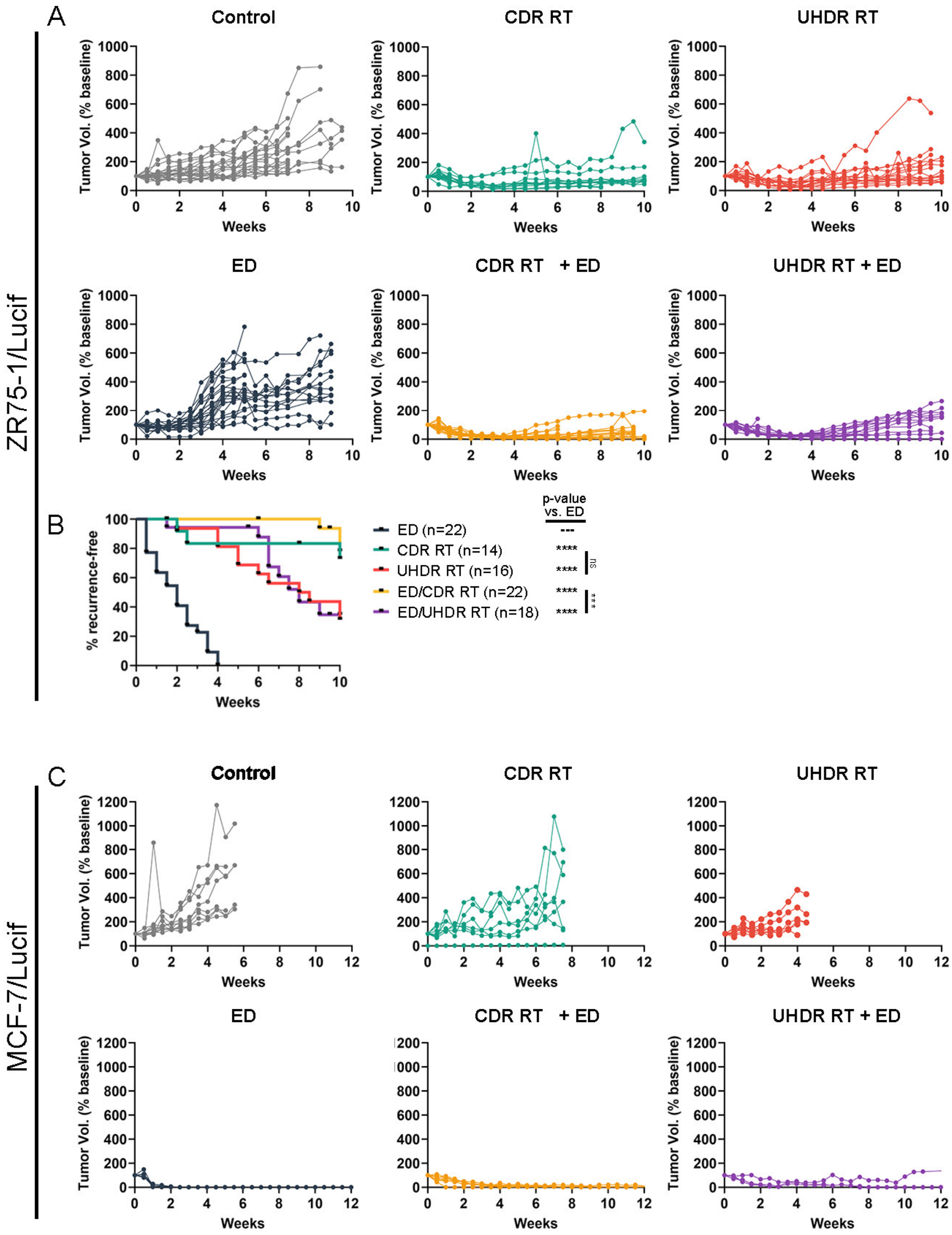

Figure S1. Individual tumor growth curves for *in vivo* studies. (A/C) Each graph depicts volumes of individual tumors within a treatment group. (B) Mice bearing ZR75-1/Luc tumors were treated as in Fig. 5 and monitored for recurrence defined as re-growth to “Day 0 volume.” Timelines of recurrence are shown in Kaplan-Meier plots. ***p≤0.001, ****p≤0.0001 by log-rank test. ns: not significant.

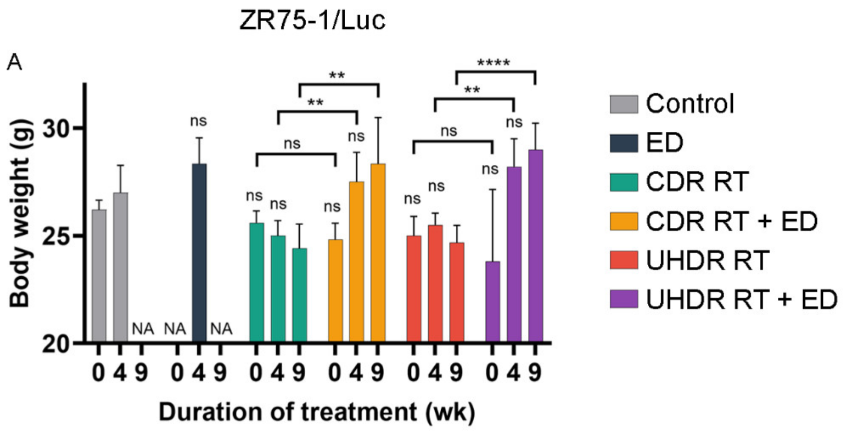

Figure S2. Body weights of mice bearing ZR75-1/Luc tumors. Weights of 5-6 mice per treatment group were recorded at baseline and after 4 and 9 wk of treatment. Data are presented as mean + SD. **p<0.01, ****p<0.0001 by t-test compared to respective time point of control group unless otherwise indicated with brackets. ns: not significant. NA: not available.
