# Supplementary material for: Combined Radiation and Endocrine Therapies Elicit Benefit in ER+ Breast Cancer
Source: Cancers (Basel). 2025 Jun 9;17(12):1921. doi: 10.3390/cancers17121921 (PMC12190792; doi:10.3390/cancers17121921)

Original blot images for Fig. 1C.

Top image (short exposure): upper blot is for NRF2 (no signal detected); lower blot is for Actin.
Bottom image (long exposure): upper blot is for NRF2; lower blot is for Actin (red indicates overexposure).

Lane	Sample
1	T47D cells grown in full medium (10% FBS)
2	T47D cells grown in HD medium (10% DCC-FBS) for 14 d
3	T47D cells grown in full medium (10% FBS), irradiated with 6 Gy CDR RT
4	T47D cells grown in HD medium (10% DCC-FBS) for 14 d. then irradiated with 6 Gy CDR RT
5	T47D cells grown in full medium (10% FBS) irradiated with 6 Gy UHDR RT
6	T47D cells grown in HD medium (10% DCC-FBS) for 14 d, irradiated with 6 Gy UHDR RT

Lysates were harvested at 1 h post-RT.

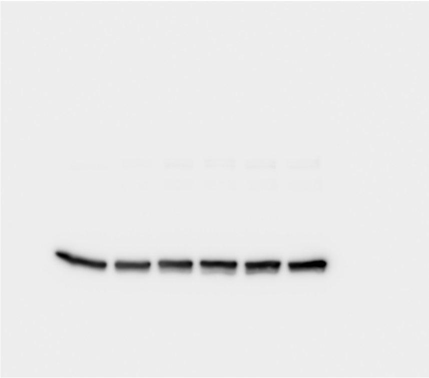

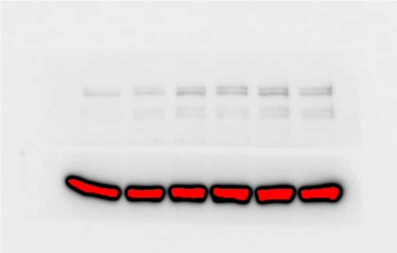

Original blot images for Fig. 1D.

Top image (short exposure): upper blot is for NRF2 (no signal detected); lower blot is for Actin.
Bottom image (long exposure): blot is for NRF2.

Lane	Sample
1	ZR75-1 cells grown in full medium (10% FBS)
2	ZR75-1 cells grown in HD medium (10% DCC-FBS) for 14 d
3	ZR75-1 cells grown in full medium (10% FBS), irradiated with 6 Gy CDR RT
4	ZR75-1 cells grown in HD medium (10% DCC-FBS) for 14 d. then irradiated with 6 Gy CDR RT
5	ZR75-1 cells grown in full medium (10% FBS) irradiated with 6 Gy UHDR RT
6	ZR75-1 cells grown in HD medium (10% DCC-FBS) for 14 d, irradiated with 6 Gy UHDR RT

Lysates were harvested at 1 h post-RT.

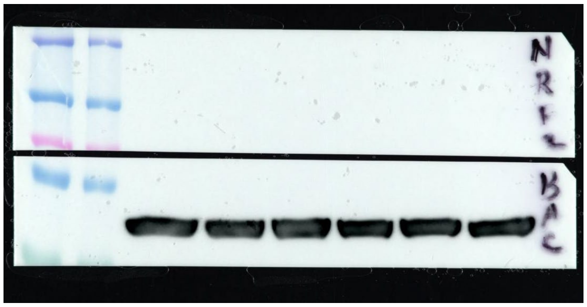

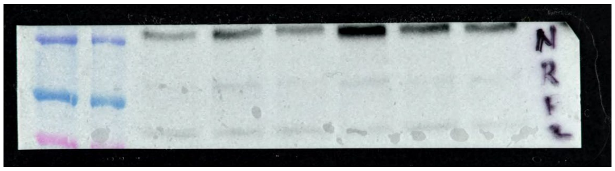

Supplement: Supplementary file 1 [file cancers-17-01921-s001.zip › File S1. Original blot images.pdf]
